# Spin canting across core/shell Fe_3_O_4_/Mn_x_Fe_3−x_O_4_ nanoparticles

**DOI:** 10.1038/s41598-018-21626-0

**Published:** 2018-02-21

**Authors:** Samuel D. Oberdick, Ahmed Abdelgawad, Carlos Moya, Samaneh Mesbahi-Vasey, Demie Kepaptsoglou, Vlado K. Lazarov, Richard F. L. Evans, Daniel Meilak, Elizabeth Skoropata, Johan van Lierop, Ian Hunt-Isaak, Hillary Pan, Yumi Ijiri, Kathryn L. Krycka, Julie A. Borchers, Sara A. Majetich

**Affiliations:** 1 Physics Department, Carnegie Mellon University, Pittsburgh, PA 15213 USA; 2 Applied Physics Division, Physical Measurement Laboratory, NIST, Boulder, CO 80305 USA; 3 Materials Science and Engineering Department, Carnegie Mellon University, Pittsburgh, PA 15213 USA; 4 Chemistry Department, Carnegie Mellon University, Pittsburgh, PA 15213 USA; 5 SuperSTEM, Sci-Tech Daresbury Campus, Daresbury, WA4 4AD UK; 6 Department of Physics, University of York, Heslington, York YO10 5DD UK; 7 The York-JEOL Nanocentre, York Science Park, Heslington, York YO10 5BR UK; 8 Physics and Astronomy Department, University of Manitoba, Winnipeg, MB R3T 2N2 Canada; 9 Physics and Astronomy Department, Oberlin College, Oberlin, OH 44074 USA; 10 NIST Center for Neutron Research, NIST, Gaithersburg, Maryland 20899 USA

## Abstract

Magnetic nanoparticles (MNPs) have become increasingly important in biomedical applications like magnetic imaging and hyperthermia based cancer treatment. Understanding their magnetic spin configurations is important for optimizing these applications. The measured magnetization of MNPs can be significantly lower than bulk counterparts, often due to canted spins. This has previously been presumed to be a surface effect, where reduced exchange allows spins closest to the nanoparticle surface to deviate locally from collinear structures. We demonstrate that intraparticle effects can induce spin canting throughout a MNP via the Dzyaloshinskii-Moriya interaction (DMI). We study ~7.4 nm diameter, core/shell Fe_3_O_4_/Mn_x_Fe_3−x_O_4_ MNPs with a 0.5 nm Mn-ferrite shell. Mössbauer spectroscopy, x-ray absorption spectroscopy and x-ray magnetic circular dichroism are used to determine chemical structure of core and shell. Polarized small angle neutron scattering shows parallel and perpendicular magnetic correlations, suggesting multiparticle coherent spin canting in an applied field. Atomistic simulations reveal the underlying mechanism of the observed spin canting. These show that strong DMI can lead to magnetic frustration within the shell and cause canting of the net particle moment. These results illuminate how core/shell nanoparticle systems can be engineered for spin canting across the whole of the particle, rather than solely at the surface.

## Introduction

Single domain, magnetic nanoparticles (NPs) have been widely investigated for applications in materials science and biomedicine^1–3^. Most research on nanoparticles has largely focused on how size, shape, structure and chemical composition affect magnetic properties^4,5^. The past decade has seen major advances in terms of sub-nanometer probes of materials and fabrication techniques, which have allowed exploration of magnetic effects due to a non-uniform spin configuration within a nanoparticle^6–9^. Core/shell nanoparticles with hard/soft magnetic layers can be synthesized with high structural precision for engineered magnetic interactions between the layers^10^. Controlled magnetic interactions between core/shell layers have been used to tailor the magnetic response of cubic nanoparticles^11^ and for enhanced heat generation for magnetic hyperthermia^12^. Particular attention has been paid to Fe_3_O_4_/Mn-ferrite core/shell structures with strong exchange coupling in nanoparticle systems^13^.

Spin canting at high fields was first proposed to explain a reduced moment in Mn ferrite found by both neutron diffraction and Mössbauer spectroscopy^14^, and later supported by high field differential susceptibility^15^ and thermomagnetization^16^ measurements. Since then numerous studies of ferrite NPs have identified a separate component in Mössbauer spectra attributed to canted spins^17–20^, or to disordered surface spins^21^. Small angle neutron scattering demonstrated spin canting that was coherent within a surface shell for Fe_3_O_4_ NPs^7^; this has been confirmed by a combination of high resolution electron energy loss spectroscopy (HREELS) and density functional theory (DFT) calculations^9^.

Fe_3_O_4_/Mn-ferrite core/shell NPs are expected to have enhanced spin canting, enabling closer examination of the detailed mechanism and the spin configuration. Surface spins of NPs are more susceptible to canting than those in the interior^7^. For Fe_3_O_4_, canting was ascribed to the competition between exchange and Zeeman interactions^22^, but many questions remain, prompting investigation of other systems. The Curie temperature of MnFe_2_O_4_, which is proportional to the exchange stiffness, is 300 °C, compared with 585 °C for Fe_3_O_4_. The bulk magnetocrystalline anisotropy is lower than that of Fe_3_O_4_^23^, unlike CoFe_2_O_4_, where the high anisotropy restricts the amount of canting^24^. Here, we study a core/shell system comprised of a Mn-ferrite shell and Fe_3_O_4_ core for a few distinct reasons. First, core-shell systems with contrast in magnetocrystalline anisotropy and exchange coupling between core and shell are becoming increasingly popular for biomedical applications. Thus, it is essential to characterize a model system with a variety of probes and techniques to demonstrate how chemical composition can alter a core/shell system’s magnetic properties on the nanoscale. Secondly, manganese ferrite has a relatively low magnetocrystalline anisotropy relative to other Fe-based ferrites. It was chosen for a study on spin canting because its internal spins will be less tightly bound to a crystal lattice and, therefore, more susceptible to canting effects. Finally, Mn-ferrite and Fe_3_O_4_ are particularly bio-compatible compared to the rest of Fe-based ferrites (for instance, Co-ferrite is quite toxic). Therefore, we chose a core/shell system comprised of two materials that have a high potential for biomedical or therapeutic application.

**Figure 1:**
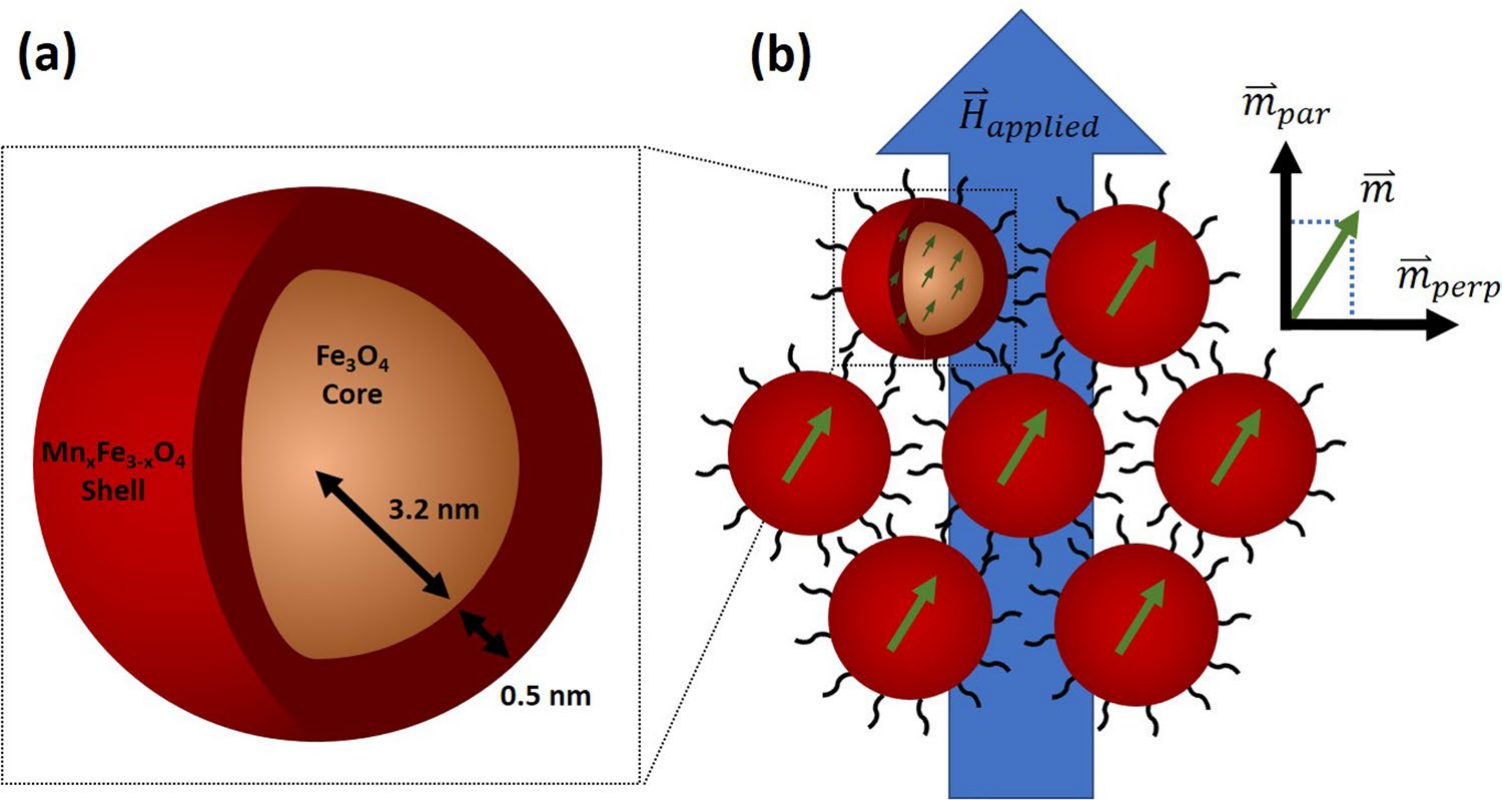
(**a**) Schematic showing chemical and physical composition of core/shell nanoparticles. (**b**) Representative figure showing coherent spin canting across nanoparticles under application of applied magnetic field. Due to interparticle correlation of canted spins, the domain has a net canted magnetization that can be measured in terms of parallel and perpendicular components. Particles are physically separated from one another due to a stabilizing, surfactant coating (oleic acid) on their surface.

The core/shell system studied here is depicted in Fig. 1a. The nanoparticles have a diameter of ~7.4 nm and a thin, 0.5 nm, Mn-ferrite shell. Here we describe the chemical and magnetic structure of the core and shell, in addition to the small angle neutron scattering results that reveal multiparticle correlations both parallel and perpendicular to the applied magnetic field. Such correlations are depicted in Fig. 1b, where the canting direction of one particle is aligned with other, nearby particles. Data are interpreted with guidance from atomistic simulations of spin configurations expected for different models of the driving forces for spin canting.

## Results

### Electron microscopy and Electron Energy Loss Spectroscopy (EELS)

**Figure 2:**
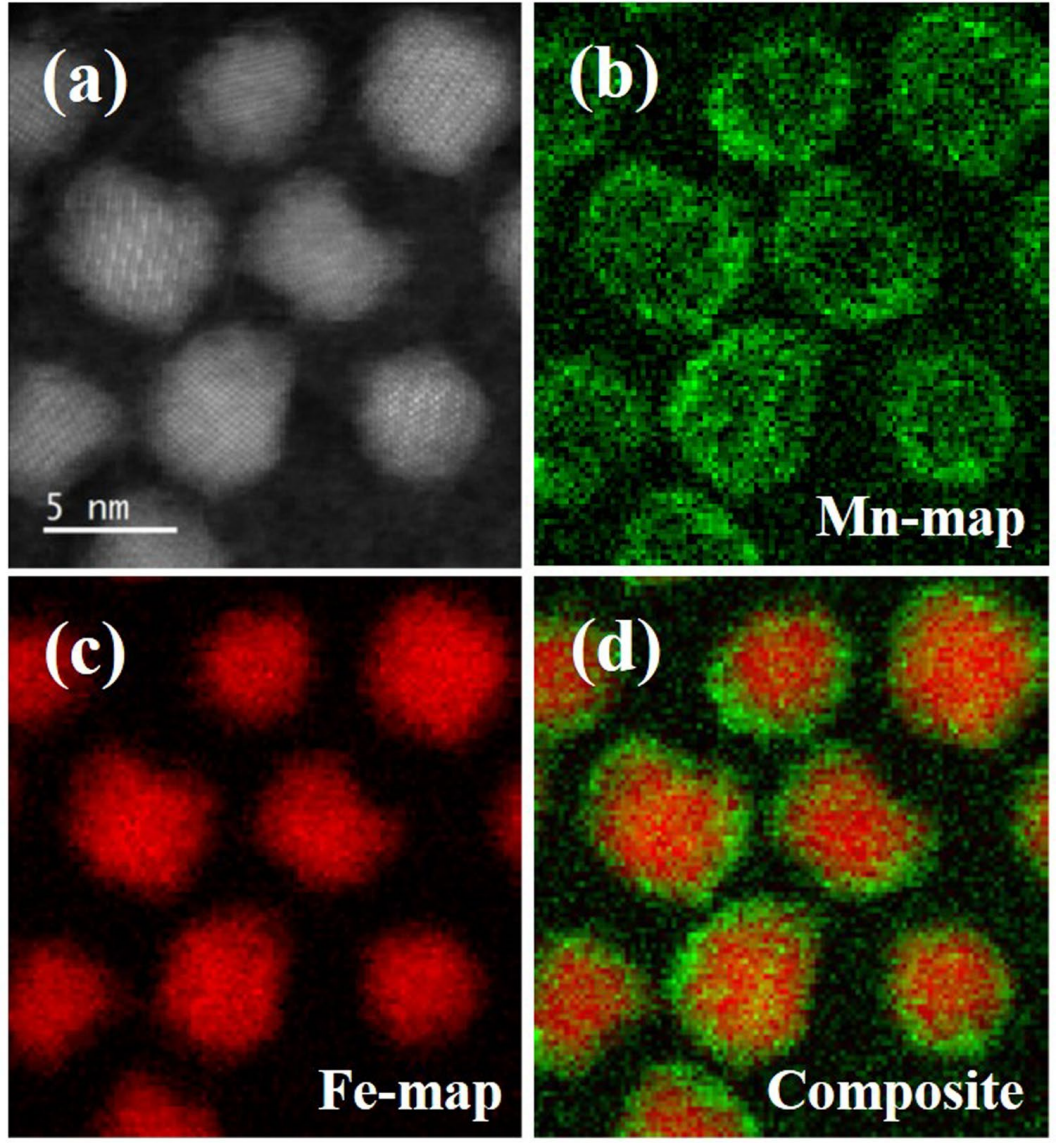
(**a**) HAADF-STEM images of nanoparticles showing spinel structure in magnetite cores. (**b**) integrated map of the Mn L_2,3_ EELS signal, where green intensity shows the Mn distribution. (**c**) integrated map of the Fe L_2,3_ EELS signal, where red intensity shows the Fe distribution. (**d**) Composite image showing Fe-rich core and Mn-rich shell in the nanoparticles.

The NPs have a core/shell morphology. In Fig. 2a, High angle annular dark field (HAADF) scanning transmission electron microscopy (STEM) shows that the particles have a crystalline, spinel core consistent with magnetite (Fe_3_O_4_), and a less structured shell, suggesting that it is disordered. In other words, imaging reveals that the surface of particles is not perfectly crystalline and appears to show a degree of disorder extending a few atomic planes from the surface. This disorder is observable in the gallery of HAADF-STEM images of particles in Supplementary Fig. S1. The EELS maps of Fig. 2b and c reveal the distribution of Mn and Fe, respectively. The composite image, Fig. 2d, indicates particles with an iron-rich core and a ~0.6 nm thick manganese-rich shell. Conventional transmission electron microscopy (TEM) was used to measure an average diameter of the nanoparticles, which was found to be 7.0 ± 1.4 nm. A shell is formed due to the large gap in decomposition temperature between Fe(acac)_3_ at 186 °C and Mn(acac)_2_ at 249 °C^25,26^. During synthesis, the two-hour heating step at 200 °C is sufficient to decompose the Fe(acac)_3_ but not high enough for Mn(acac)_2_ decomposition. When the temperature is raised to reflux at 300 °C, magnetite cores nucleate and grow first, followed by growth of the Mn ferrite shells. In some cases core/shell NPs have been prepared by design with a two-stage process^8,13,27^, while in other some cases the differential decomposition may have unintentionally generated core/shell NPs^20,28,29^.

### Chemical composition

**Figure 3:**
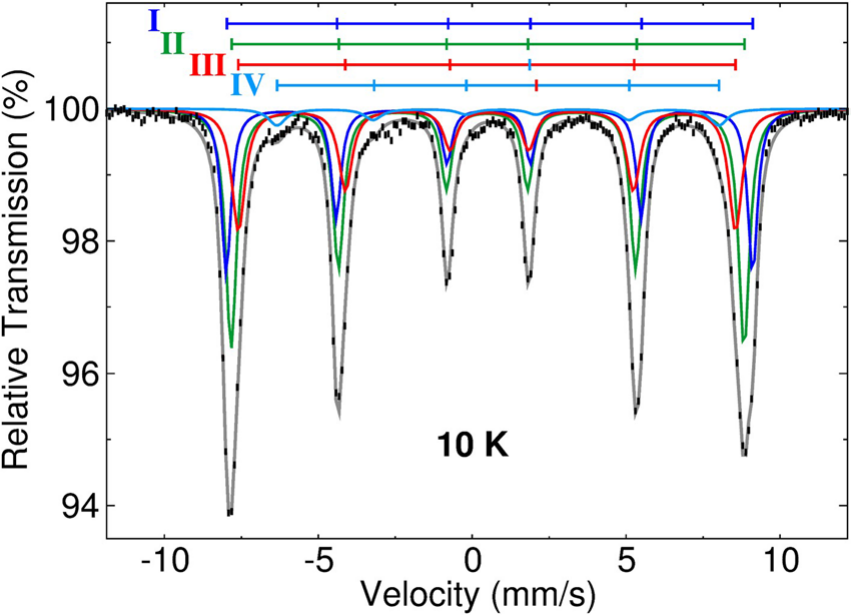
Mössbauer spectrum of core/shell nanoparticles measured at 10 K. Subspectra (I − IV) describe the simulated Fe-sites within the core and shell as described in the text and Supplementary Table S2.

While there are gradations of composition and the interfaces are not abrupt, a simplified model is useful in interpreting the magnetic behavior of the core/shell NPs. The average compositions of the core and shell were determined from a combination of the electron microscopy results with atomic absorption spectroscopy (AAS), Mössbauer and X-ray absorption spectroscopy (XAS) and X-ray magnetic circular dichroism (XMCD). From AAS, the molar ratio of Fe:Mn for the entire NP was 4.75:1, with an error of ±2% which deviates significantly from the 2:1 ratio for the starting materials. A 4:1 ratio was reported for Mn ferrite-like nanoparticles prepared in a similar manner^30^. Figure 2d suggests a magnetite-like core and a manganese ferrite-like shell, but to specify the stoichiometry, it is necessary to know the ionic charge and site occupancies. Mössbauer spectroscopy provides information about the Fe atoms via their hyperfine fields (*B*_*hf*_) that describe the local magnetic environments, and the isomer shifts (*δ*) and quadrupole splittings (*Δ*) that detail the local electronic environments. The measured asymmetric six-line spectrum (Fig. 3) indicates multiple Fe-sites, as expected for a spinel ferrite. The fitted hyperfine parameters (Supplementary Table S2) are typical for the Fe^3+^ A-site (tetrahedral), and Fe^2+^ and Fe^3+^ B-sites (octahedral) values in Fe_3_O_4_, as well as octahedral Fe-sites of a manganese ferrite^5,14^. Specifically, as shown in Fig. 3, the Fe^3+^ A-, and Fe^2+^ and Fe^3+^ B-sites of Fe_3_O_4_ are described by subspectra I, II, and III, and the shell Fe-sites of Mn_x_Fe_3−x_O_4_ are described by subspectra II and IV. The significantly lower *B*_*hf*_ and broadened linewidth of site IV is due to a large number of Mn nearest neighbors^14^ and is consistent with Fe-surface sites experiencing strongly reduced exchange strengths due to Mn^3+^ B-site neighbors (discussed below). The Mössbauer spectrum fit results also indicate that the Mn_x_Fe_3−x_O_4_ phase contains no Fe A-sites, and the Fe B-sites have predominantly 3+ charges. Since the area of each spectral component provides a direct measure of the occupancy of the relevant Fe site, given that the recoil-free fraction of the A- and B-sites is equal at 10 K^31^, an initial estimate of the core/shell nanoparticle stoichiometry would be it having a pure Fe_3_O_4_ core and a Mn_x_Fe_1−x_O_4_ shell with x >0.8^32^.

**Figure 4:**
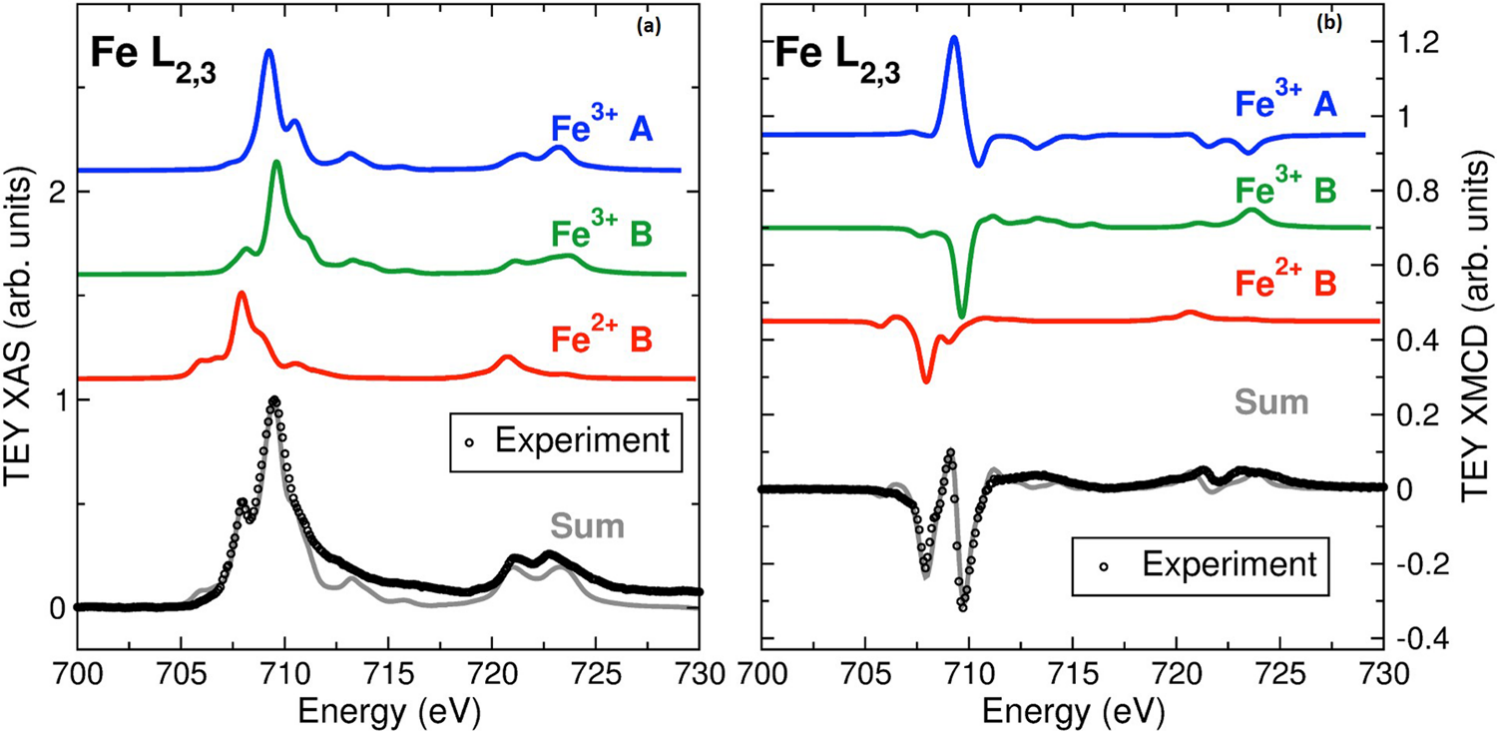
Calculations of (**a**) XAS and (**b**) XMCD of Fe^2+^ B-, Fe^3+^ A-, and Fe^3+^ B sites, along with the experimental total electron yield (TEY) data at 10 K with ±1 T. The experimental spectra are shown in comparison with a weighted sum of calculated spectra (26% Fe^3+^ A-, 31% Fe^2+^ B-, and 43% Fe^3+^ B-site).

**Figure 5:**
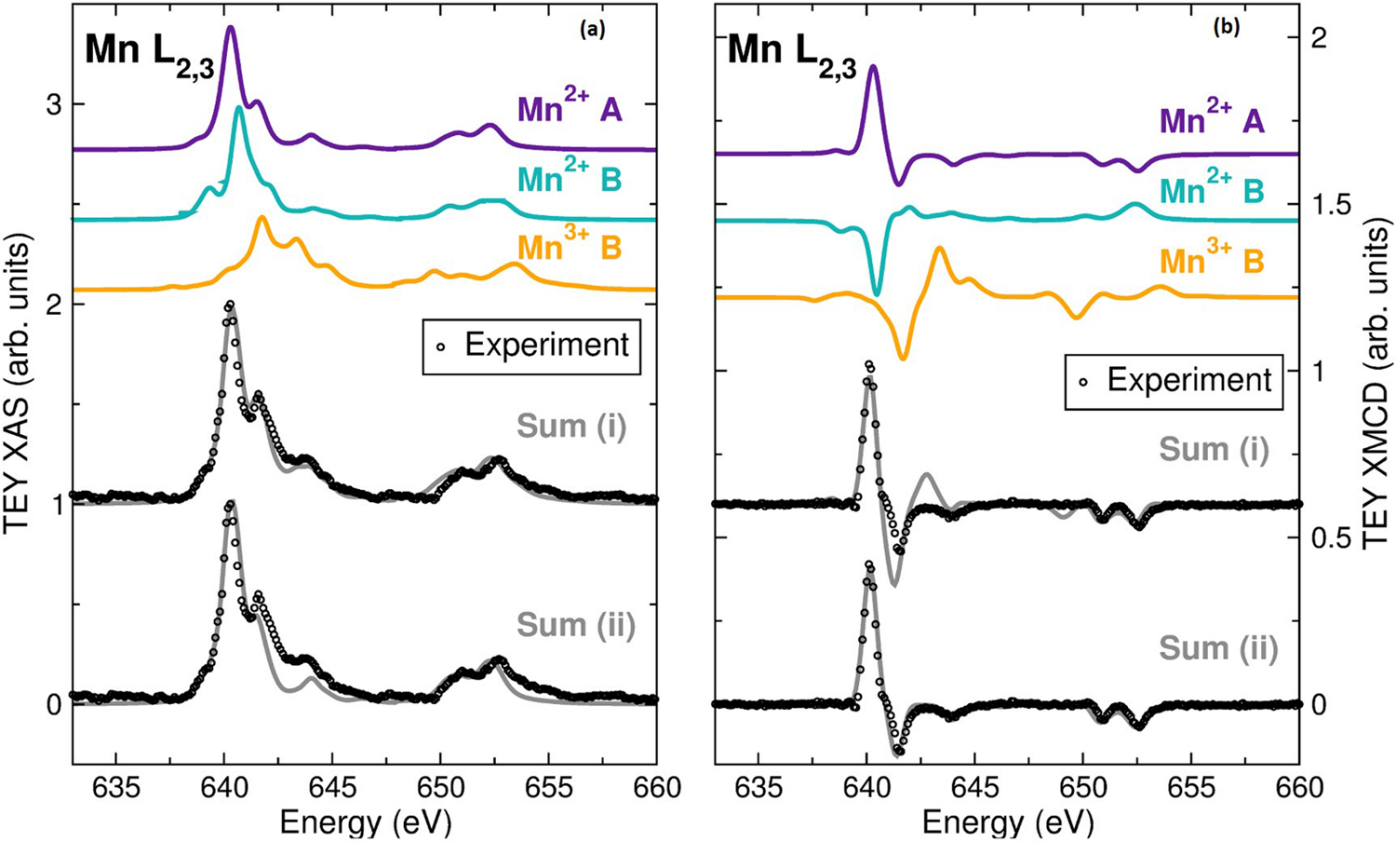
Calculations of (**a**) XAS and (**b**) XMCD of Mn^2+^ A-, Mn^2+^ B-, and Mn^3+^ B-sites. Also shown is a comparison of the XAS measured at 75 K with the sum of the calculated spectra of (i) 62% Mn^2+^ A-, 16% Mn^2+^ B-, and 22% Mn^3+^ B-sites, and (ii) 80% Mn^2+^ A-, 20% Mn^2+^ B-sites. (**b**) XMCD of Mn^2+^ A-, Mn^2+^ B-, and Mn^3+^ B-sites and a comparison of the XMCD measured at 10 K in ±1 T with the sum of (i) 62% Mn^2+^ A-, 16% Mn^2+^ B-, and 22% Mn^3+^ B-sites, and (ii) 80% Mn^2+^ A-, 20% Mn^2+^ B-sites.

XAS and XMCD experiments were used to identify clearly the configuration of the Mn sites and (in combination with the Mössbauer spectrum analysis) unequivocally ascertain the Fe site relationships and to identify the nature of magnetic coupling of the various Fe and Mn ions. The Fe XAS and XMCD in Fig. 4 identify the combination of Fe^3+^ A-, and Fe^2+^ and Fe^3+^ B-site ferromagnetic A-A and B-B, and antiferromagnetic A-B superexchange coupling of the spinel ferrites, which is consistent with the Mössbauer spectroscopy analysis. Notably, the Fe XMCD differs quite significantly from the spectrum of a ferromagnetic single-phase Fe-oxide (Fe_3_O_4_ or γ-Fe_2_O_3_); the three-peak structure at the Fe *L*_3_-edge (between 705 and 715 eV) is indicative of the nanoparticles presenting a significantly lower Fe A-site occupancy than a typical Fe-oxide. However, the Fe A-site occupancy is much larger than that of MnFe_2_O_4_^33–35^. This result is consistent with the multiple phases indicated by TEM and identified by the Mössbauer spectroscopy. Furthermore, the Mn XAS and XMCD of the core/shell nanoparticles (shown in Fig. 5) also present a clear departure from the Mn-spectra of Mn_3_O_4_, which displays a substantially broadened lineshape at the Mn L_3_-edge centered at 640 eV; instead, the data show a signature that is consistent with the presence of a Mn_x_Fe_3−x_O_4_ shell^35,36^.

To quantify the site occupancies, the measured XAS and XMCD *L*_3_-edge regions of the spectra were modeled using ligand field multiplet calculations of the individual Fe and Mn sites^37,38^. The Fe XAS and XMCD spectra are best described by 26% Fe^3+^ in A-sites, and B-site populations of 31% Fe^2+^ and 43% Fe^3+^ (solid lines in Fig. 4). Agreement between the XAS and XMCD site assignments is a clear indication that all these sites contributed to the magnetism. The Mn XAS is best described by 62% Mn^2+^ in A-sites, and 16% Mn^2+^ with 22% Mn^3+^ in B-sites (solid lines in Fig. 5a). By contrast with the Fe-site magnetism, the Mn XMCD spectrum is best described by 80% Mn^2+^ A-site and 20% Mn^2+^ B-site magnetism (Fig. 5b). This suggests strongly that the Mn^3+^ sites do not have a net magnetization. Additionally, the magnetic Mn site magnetizations are aligned with ferromagnetic A-A and B-B couplings to the Fe sites, and antiferromagnetic A-B couplings, fully consistent with a spinel Mn_x_Fe_3−x_O_4_.

Based on the structural and chemical characterizations that quantify the relative Fe and Mn sites and provide the site valence for the entire nanoparticle, using the known nanoparticle total size (from TEM), and the Fe and Mn elemental abundances obtained from atomic absorption spectroscopy (that leads to a relative Fe-oxide and Mn_x_Fe_3−x_O_4_ phase fraction), we determine that the nanoparticles consist of a 6.4 nm Fe_3_O_4_ core and a 0.5 nm thick Mn_x_Fe_3−x_O_4_ shell with *x* ∼ 1.0 and an ion distribution of (Mn^2+^)[Fe^2+^_0.38_ Fe^3+^_1.α_ Mn^2+^_0.25_ Mn^3+^_0.35_]O^2−^_4_. The Mn_x_Fe_3−x_O_4_ shell thickness estimated from spectroscopic measurements is consistent with the results from EELS mapping. This formula for the ion distribution indicates that the Mn_x_Fe_3−x_O_4_ has a slight overabundance of B-sites (B/A = 2.23 versus a maximum of 2 for the spinel structure). It is likely that the Mn^3+^ B-sites are clustered at the surface^39^ and are not well exchange coupled to the other sites, accounting for the lack of a Mn^3+^ XMCD spectral component. Furthermore, Mn surface clustering is consistent with the very small amount of Fe site IV observed in the Mössbauer spectrum that is attributed to Fe ions at surface sites affected by nearby surface Mn sites.

### Magnetic properties and spin configuration

**Figure 6:**
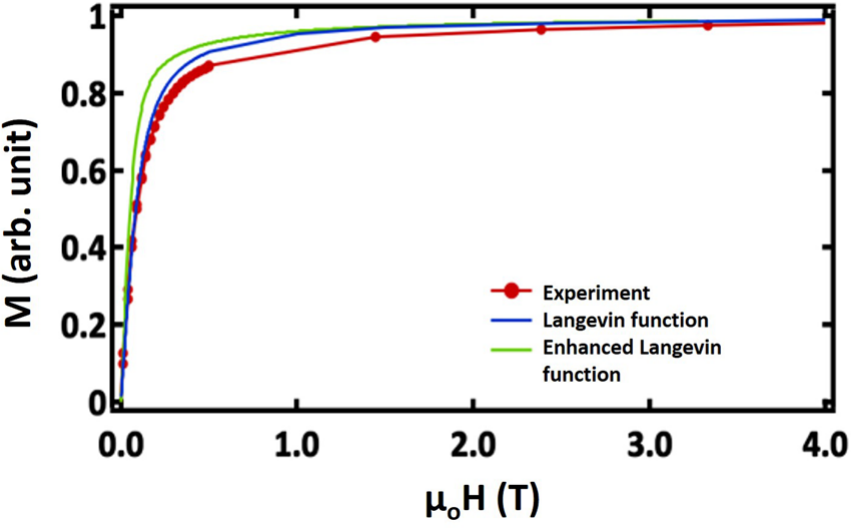
Magnetization and magnetic correlations in the core/shell NPs. (**a**) Magnetization curve at 200 K for a dilute dispersion of the nanoparticles, compared with both the standard and a modified^40^ Langevin function, using the independently determined value of *μ*.

Figure 6 shows the magnetization curve for a dilute dispersion of the core/shell NPs at 200 K. Here, preparation of a dilute dispersion indicates that the particles were diluted by a factor of 100 with toluene and dispersed in liquid eicosane at a temperature just above room temperature (melting point of eicosane 36 °C). When the sample is brought back to room temperature, the eicosane sets, and then particles are immobilized in the waxy matrix at distances exceeding a few particle diameters. This ensures that interparticle, magnetic interactions do not influence the magnetization of particles. The results for different temperatures are given in Supplementary Fig. S3. The 300 K hysteresis loop has no coercivity, while the 10 K loop shows a small coercivity of ~150 Oe. Magnetization curves measured after cooling in a 9 T field showed no evidence of a loop shift, as would be expected due to exchange bias. The zero field-cooled magnetization indicated a blocking temperature of 15 K (Supplementary Fig. S4a). Dense assemblies of nanoparticles showed a higher blocking temperature (Supplementary Fig. S4b), indicating that the dilute assemblies in eicosane effectively reduced interparticle interactions. With Fe_3_O_4_ core/Mn_3_O_4_ shell NPs^27^, zero field-cooling revealed a change near the magnetic ordering temperature of Mn_3_O_4_ (43 K), but no similar feature was observed here.

At first glance, the core/shell NPs appear to show typical behavior of a soft magnetic material, but closer inspection of Fig. 6 reveals more complex behavior. For assemblies of noninteracting superparamagnetic NPs, the magnetization curve is often fit to a Langevin function to determine the average NP moment, *μ*. Here *μ* was determined independently from a combination of the TEM size distribution, the Fe and Mn concentrations found by atomic absorption, and the total sample magnetic moment measured from a SQUID magnetometer. For a single particle, *μ* was found to be 5.88 × 10^−20^ Am^2^. A Langevin function of the following form was used to generate M(H) curves for single domain nanoparticles,

1 $$M(H)=n\mu \,L(\frac{{\mu }_{o}H\mu }{{k}_{B}T})$$

where *n* is the concentration of particles, *μ* is the particle moment, *H* is the applied field, *k*_*B*_ is the Boltzmann constant, and *T* is temperature. The Langevin function, *L(x*) = 1/tanh(*x*) − 1/*x*.

*M(H)* curves generated using this value of the particle moment show deviations below 2 T, relative to the experimental data, and also to a modified Langevin model that includes anisotropy effects^40^. If the particles were uniformly magnetized, then a single value of *μ* would fit both the high and low field magnetization curve. Here the low field data appears to have reduced *μ* parallel to the applied field. In particular, we see a reduction of ~15–20% of the experimental, in-plane moment at a field of 0.1 T. Since magnetization has not been added or subtracted from the system, it is likely that the reduction observed in *M(H)* data is due to canting of spins within nanoparticles axes perpendicular to the applied field direction.

**Figure 7:**
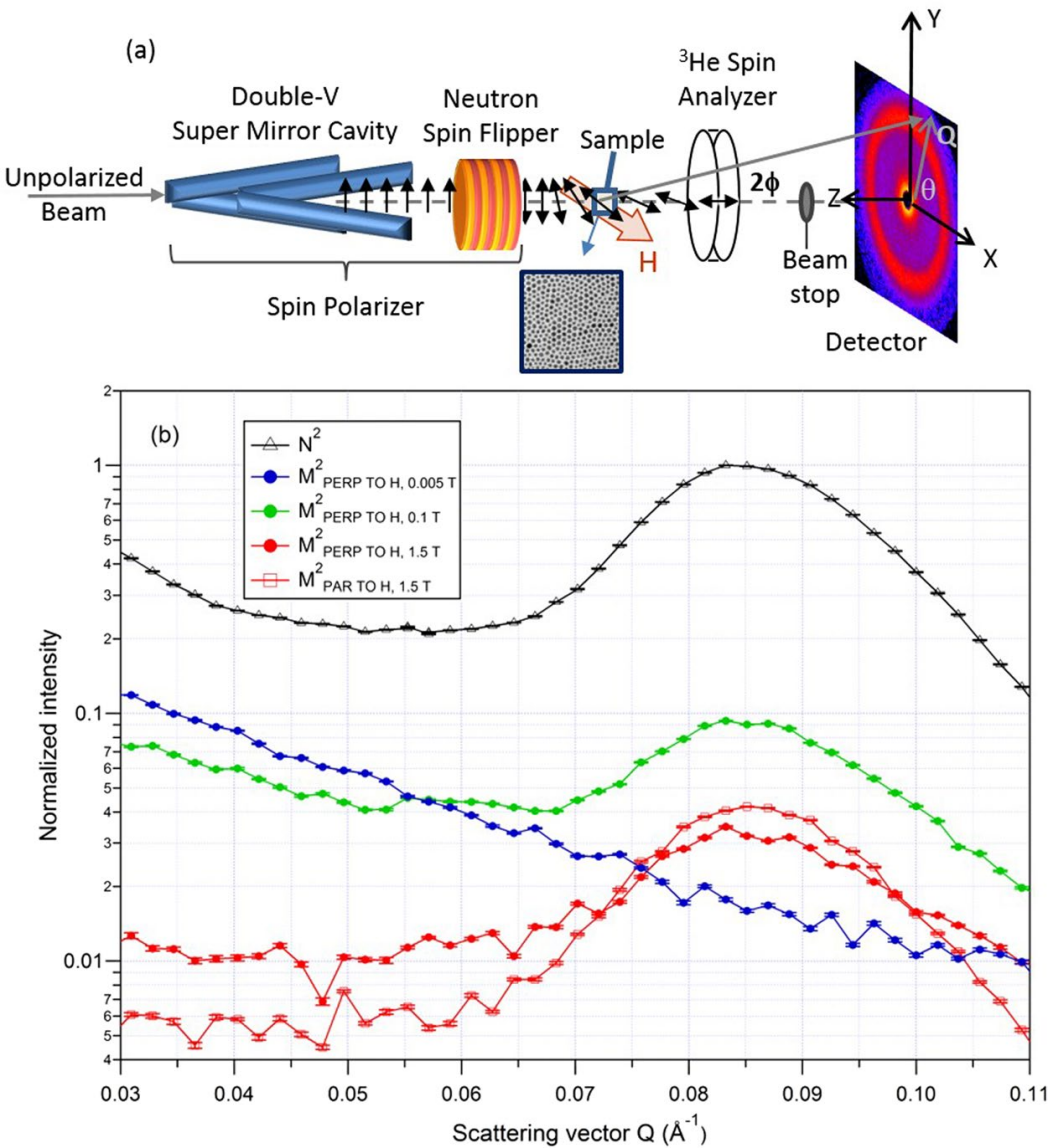
(**a**) Schematic of PASANS measurement process and (**b**) PASANS intensity data on dense, crystallized assemblies of the core/shell nanoparticles as a function of scattering vector Q for 200 K and a variety of applied magnetic field (H) conditions. Note that the magnitude of |**Q**| = 4πsin(ϕ/λ) where 2ϕ is the scattering angle between the source and detector and λ is the neutron wavelength. Data are normalized against the unchanging peak of the structural scattering (N^2^) with the N^2^, M^2^_PERP_, and M^2^_PAR_ components extracted as described in the Supplementary Data Note S5, using sector averages of the 2D data, with θ defined as the angle from Q in the detector plane to the field (or X) axis.

Polarization-analyzed small-angle neutron scattering (PASANS) measurements^41,42^ were performed to further explore the unusual magnetic behavior of these particles, in this case organized into ordered dense assemblies. Figure 7a shows a schematic of the experimental setup in which the neutron beam was polarized either spin up (↑) or down (↓), scattered off the sample in a given field and temperature condition, and then spin-analyzed to measure all four possible scattering cross-sections (↑↑, ↑↓, ↓↑, or ↓↓). As described in the Methods section and Supplementary Data Note S5, these data were used to extract Fourier transform components sensitive solely to the structural features (*N*) or magnetic features relative to an applied magnetic field (*M*_*PAR*_ or *M*_*PERP*_).

In Fig. 7b, the structural scattering intensity (*N*^2^ in black open triangles) for the core/shell NPs displays a prominent Bragg peak centered at roughly 0.085 Å^−1^. This behavior is consistent with the resolution-smeared primary reflections expected for face-centered cubic (FCC) crystallites and observed in similar dense assemblies of Fe_3_O_4_ or CoFe_2_O_4_ nanoparticles^7,24^. The Bragg peak corresponds to an FCC lattice parameter of ~12 nm, consistent with close packing of ~7 nm diameter nanoparticles with a molecular, surfactant layer surrounding them. For the core/shell NPs in high magnetic field (1.5 T) and moderate temperature (200 K), the Bragg peak is observed in the magnetic scattering component related to moments parallel to the field and coherent with the structural order (*M*^2^_*PAR*_ in red open squares).

Unexpectedly, the Bragg reflection is *simultaneously seen* in the magnetic scattering component related to moments *perpendicular* to the field (*M*^2^_*PERP*_ in solid red circles). While absent in a remanent field of 0.005 T at 200 K (solid blue circles), the peak persists in a moderate field of 0.1 T (solid green circles), along with additional scattering at lower scattering vector Q. Taken together with the *M*^2^_*PAR*_ data, these *M*^2^_*PERP*_ measurements indicate that in appropriate conditions, magnetic correlations persist from nanoparticle to nanoparticle and denote coherent spin canting across *multiple* particles in the ordered assembly. A more detailed analysis of the SANS results as a function of field and temperature will be reported elsewhere.

Note this behavior contrasts markedly from that observed for Fe_3_O_4_ or CoFe_2_O_4_ nanoparticle systems^7,24^. For 9 nm diameter Fe_3_O_4_ nanoparticle assemblies, the *M*^2^_*PERP*_ measurement in large field showed a dip in the vicinity of the structural Bragg peak scattering vector, associated with the form factor of a single uncorrelated shell. The canted shell was observed to grow and shrink with field and temperature but with limited evidence of coherence from nanoparticle to nanoparticle. For 11 nm diameter CoFe_2_O_4_ nanoparticle assemblies in a large field, the perpendicular magnetic scattering showed single particle scattering, indicating an overall tilt of the magnetization that was again not coherent from particle to particle^24^. There was no evidence of a shell formation, consistent with the strong magnetocrystalline anisotropy of CoFe_2_O_4_.

## Discussion

There are still many questions concerning the origin of the spin-canted state in ferrite NPs, and the new results for the core/shell NPs offer some important insights. From Supplementary Fig. S8, the magnetization doesn’t change between much 2 and 9 T, suggesting a very stable “saturated” state for dilute particles. There is still some high field susceptibility, though, suggesting slight change in magnetization at high fields. Polarized SANS and high resolution EELS detection of spin canting were done at or below 2 T, and it is possible that spin canting is an intermediate field phenomenon, analogous to the spin-flop phase observed in some bulk antiferromagnetic oxides^43^. While there have been numerous reports of spin canting at high fields (4–6 T) based on Mössbauer spectroscopy, the NPs studied there also had considerable surface disorder. NPs with surface structural disorder can have open hysteresis loops even at 10 T, and their magnetic configuration has been modeled as a surface spin glass^44^. Though the core/shell NPs described here have a structurally disordered shell, the SANS results show coherence in the canted spin signal across multiple particles, which would not be expected for a spin glass. While our current data do not provide direct information regarding the radial distribution of the canted spins, there are many different reasons that could make surface spins more susceptible: weaker Heisenberg exchange due to a reduced number of superexchange interactions and/or lattice distortions, and Dzyaloshinskii-Moriya interactions^45,46^ or surface anisotropy associated with symmetry breaking.

**Figure 8:**
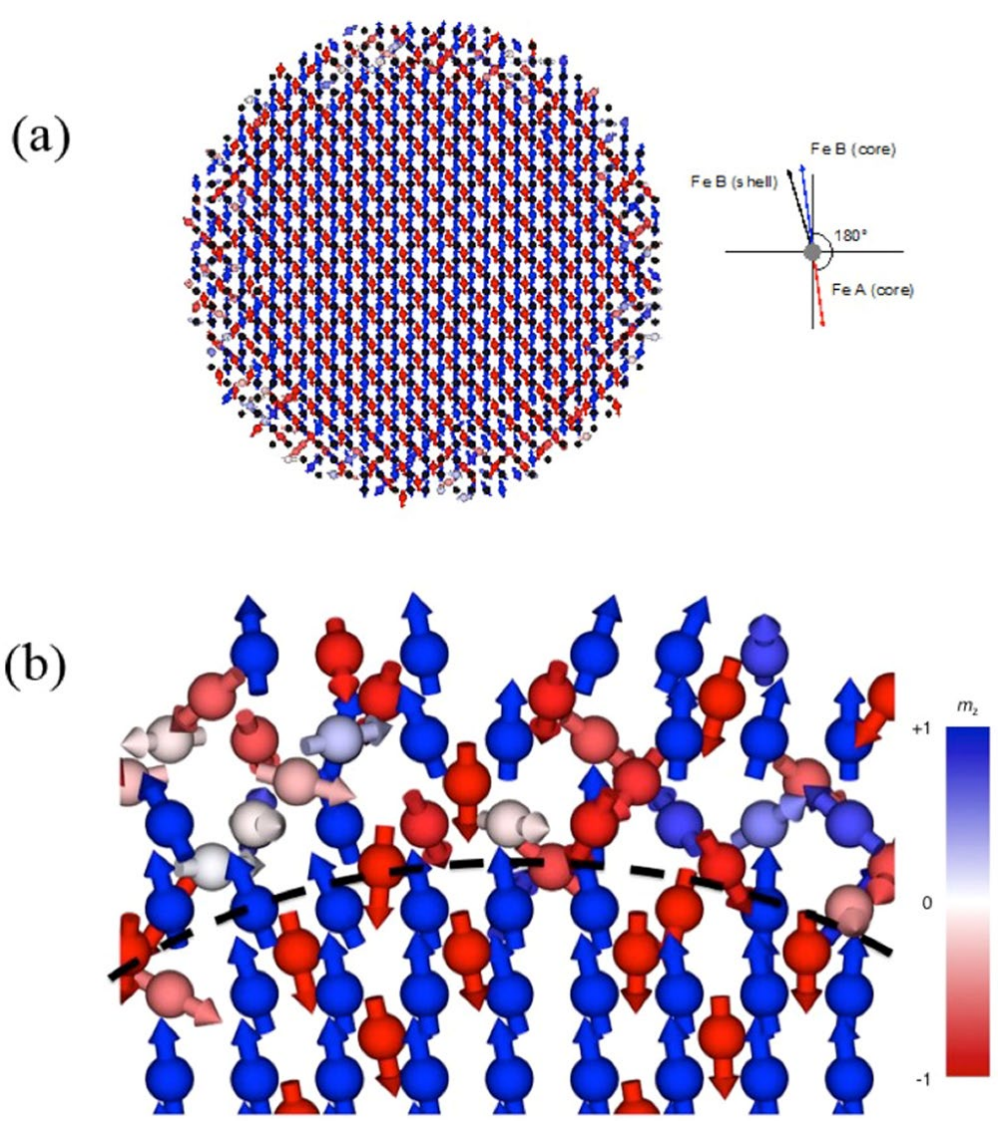
(**a**) Visualization of the simulated spin configuration of a Fe_3_O_4_ core and Mn(Fe_1−x_Mn_x_)_2_O_4_ shell including Dzyaloshinskii-Moriya interactions on Mn B sites. The simulation temperature is set at 0 K in a 0.1 T externally applied field along the [001] crystal direction. The shell shows significant local disorder due to the strong effect of the DMI on the octahedral Mn sites with *D* = *J*_*ij*_ and particularly at the surface. The frustration leads to canting of the net magnetization of the NP from the field direction of around 12 degrees, similar to an effective anisotropy. The surface frustration also gives a small intersublattice canting of the octahedral Fe sites in the shell of around 1.5 degrees compared to the core. In a NP assembly this could lead to coherent canting after field cooling, and in large fields the particle canting is significantly reduced. (**b**) Enlargement of a region of Fig. 8a, with dashed line to show the boundary between core and shell. Color bar indicates direction of spin magnetization (blue, +1 or red, −1) on the spin sites.

Atomistic spin dynamics simulations (described in Supplementary Data Note S6) were performed in order to understand the mechanisms responsible for the experimental findings. The model nanoparticle had a 5.6 nm Fe_3_O_4_ core and 0.7 nm thick MnFe_2_O_4_ shell, both with bulk-like structural and magnetic parameters. While the simulations did not identify a unique origin, some possibilities can be ruled out. An inverse spinel crystal structure was used for the crystal symmetry in the simulations, guided by the atomic detail of HAADF-STEM images. A single nanoparticle constructed for atomic simulations is depicted along the [100] zone axis in Fig. 8a. The crystal structure mirrors the nanoparticles observed with HAADF-STEM, also oriented along the [100] zone axis, in Fig. S1a, and reflects the chemical model particle depicted in Fig. 1a.

The first set of simulations considered the effect of a reduced exchange coupling between the core and shell. This means the exchange constant coupling spins at the interface of the core and shell was reduced. Here the experimental magnetization curve could be replicated, with a relative reduction of the magnetization at low fields compared to a single phase particle of the same size. However, this required that there be *no* exchange between the core and shell, so that the shell had no remanence at zero field. If the coupling were only one percent of the bulk Heisenberg exchange, this would be sufficient to align the magnetization of the core and shell at 300 K. In addition, the surface spin configuration was disordered rather than canted, as shown in Fig. S7. In a nanoparticle assembly there would be strong magnetostatic interactions among particles, but the interaction fields would not be strong enough to explain deviations that persist above 1 T. Therefore, the experimental results are unlikely to be due solely to reduced coupling between core and shell. Similarly, reduced exchange *within* the shell could not explain perpendicular multiparticle correlations.

Previous reports have suggested that the Dzyaloshinskii-Moriya Interaction (DMI) may be responsible for surface canting^9^, and therefore the second set of simulations explored this possibility. The equations describing this interaction are presented in Supplementary Data Note S6. Here 20% of the B sites in the shell are replaced by Mn and given strong DMI when interacting with the Fe B sites. For strong DMI the core Fe B sites are frustrated as they are strongly exchange coupled to the shell while the Mn B sites prefer spiral order, and a minimum energy is found when the shell cants at an angle of 1.5 degrees to the core as shown in Fig. 8a,b. There is also a more significant canting of the net particle magnetization of 12 degrees from the field direction due to the surface frustration, appearing as an effective anisotropy. This is consistent with the *M(H)* data presented in Fig. 6, since a canting angle of 12 degrees corresponds to a ~20% enhancement of the out-of-plane moment (*sin*(12°) = 0.21). This canting is significantly reduced in larger fields to 7 degrees at 2 T and 4 degrees at 10 T. In nanoparticle assemblies with dipolar interactions, this could lead to the uniform canting among the nanoparticles that is sustained to high fields. Therefore, it is feasible that DM interactions may be responsible for surface spin canting in addition to the net canting observed across the NP. Due to the structural disorder and chemical complexity of the experimental core/shell nanoparticles, other mechanisms such as local surface strains cannot be ruled out. Future work will calculate the value of the DM exchange from first principles, but for single-phase, crystalline particles.

When data sets from magnetometry, PASANS and atomistic simulations are considered together, a more complete picture of spin canting in these particles is established. The magnetization curve, which measures the magnetization parallel to the field, shows an anomalous reduction of the particle moment at moderate fields, ~15–20% at 0.1 T. PASANS data, which detect both parallel and perpendicular component, shows that at the same field and temperature conditions (0.1 T and 200 K), a significant perpendicular component in the magnetization appears on length scales commensurate with the particle spacing. This indicates that the magnetic spins of particle cant away from the direction of applied field, and that the vector magnetization is correlated between neighboring particles. One may conclude that the close packing of particles in the PASANS sample (preparation outlined in methods) indirectly induces spin canting via dipolar or interparticle effects. However, the magnetometry was performed on dilute samples also shows a significant canting effect. Atomistic simulations show spin canting in of single NPs, and offer hints about its origin. Reduced exchange, either between the core and shell or within the shell, is not significant enough to cause the degree of canting observed experimentally. The addition of a strong DMI interaction to the simulation Hamiltonian generates a strong canting effect (~20%), which is on the order of what is observed experimentally.

In summary, nanoparticles with a magnetite core and manganese ferrite shell have canted spins in moderate fields. In ordered assemblies, there are multiparticle correlations both parallel and perpendicular to the applied field. Atomistic simulations reveal that magnetic frustration in the shell, which may originate from DM interactions of Mn B site ions, leads to a modest amount of surface canting, which can act as a source of anisotropy. Strong exchange coupling between the core and shell causes the core spins to cant, as well. In dense assemblies, magnetostatic interactions among the particles favor canting of the particle moments in the same direction. This coherent canting results in a canted superferromagnet or canted supermagnet, a nanoparticle composite that collectively shows canted ferromagnetic behavior^47^. While these core/shell nanoparticles are highly complex, a similar spin canting mechanism may be responsible for variations in performance seen in nanoparticles used for magnetic hyperthermia. Surface frustration due to either DMI or local strains could affect the response of entire particles to AC magnetic fields, which would impact the heat generation. Specifically, tailoring the magnetic response and magnetization reversal in the particle should be possible by precisely tuning the DM interaction between the core and shell.

## Methods

### Synthesis

The core/shell nanopar ticles were synthesized using high temperature decomposition of metal acetylacetonate (acac) precursors with 1,2-hexadecanediol in the presence of oleic acid and oleylamine^29,48^. 2 mmol Fe(acac)_3_, 1 mmol Mn(acac)_2_, 6 mmol oleic acid, 6 mmol oleylamine, 10 mmol 1,2-hexadecanediol and 20 ml benzyl ether (>99%) were sealed in a 250 ml three-neck round-bottom flask and magnetically stirred under inert atmosphere. All products were purchased from Sigma Aldrich (Any mention of commercial products is for information only; it does not imply recommendation or endorsement by NIST). The reaction flask was heated to 200 °C and left at temperature for 2 hours under flowing Ar, then heated to 300 °C and held at reflux for 1 hour before cooling. The nanoparticles were washed three times using flocculation by ethanol, centrifugation, and redispersion in toluene.

### Atomic Absorption Spectroscopy

Atomic absorption spectroscopy (AAS) was used to determine the average Fe:Mn ratio in the nanoparticles. 125 µl of the as-made NPs dispersed in toluene were completely dissolved in 1 ml of 70% concentrated HNO_3_ and then diluted with 50 mL deionized water. To determine the concentration of Fe in solution, the absorbance from a Fe hollow cathode lamp (252.3 nm) was measured and compared with that for 2 ppm, 4 ppm, 7 ppm, and 10 ppm Fe standard solutions (Sigma Aldrich). A similar approach with a Mn standard and Mn hollow cathode lamp (279.8 nm) was used to determine the Mn concentration.

### Electron Microscopy

A JEOL 2000 EX transmission electron microscope was used to evaluate shape, monodispersity and diameter of the NPs. The structural and chemical composition of the NPs were characterized with a Nion UltraSTEM-MC ‘Hermes’ aberration corrected scanning tunneling microscope (STEM), equipped with a Gatan Enfinium spectrometer (Any mention of commercial products is for information only; it does not imply recommendation or endorsement by NIST). The microscope was operated at 100 kV and the optics were configured to form a ∼0.9 Å probe with a convergence semi- angle of 30 mrad and a probe current of ~110 pA. The inner and outer collection semi-angles for high angle annular dark field (HAADF) imaging were 86 and 190 mrad, respectively. The native energy spread of the cold field electron source was 0.3 eV and EELS data were acquired at spectrometer dispersions of 0.5 eV/channel, yielding an effective energy resolutions of 1.5 eV respectively, while the EELS collection semi-angle was set to 60 mrad. Spatially-resolved EELS measurements were performed by rastering the electron probe serially across a defined region and collecting an EEL spectrum at each point. The EELS data were de-noised by Principle Component Analysis using the CiMe-plug-in for Digital Micrograph^49^. Chemical maps are then created by integrating at each point of these spectrum images the spectrum intensity over an ∼40 eV window above the Mn *L*_*2,3*_ and Fe *L*_*2,3*_ EELS edge onsets after background subtraction using a power law model. Example spectra are depicted in Fig. S9.

### Mössbauer Spectroscopy (MS)

The transmission Mössbauer spectrum was collected on NP powder at 10 K using a WissEl constant acceleration spectrometer with a 10-GBq ^57^CoRh source. The source drive velocity was calibrated using a 6-μm thick α-Fe foil at room temperature. The Mössbauer spectrum was described using a 1^st^-order perturbation of the nuclear Hamiltonian, appropriate when the hyperfine field energy is significantly larger than the electric field gradient (as is the case for all ferrites). Spectral lineshapes (that incorporate the lifetime of the excited state, and describe small amounts of disorder through broadened FWHM Lorentzian linewidths) and the transition probabilities (that map onto the site occupancy assignments) were calculated for a powder average system, in keeping with the sample used for spectroscopy. Fit parameters (listed in Supplementary Table S2) including the spectrum baseline were established using non-linear least squares regression analysis where the cross-correlations were minimized.

### X-ray absorption spectroscopy (XAS) and x-ray magnetic circular dichroism (XMCD)

XAS and XMCD measurements were done at beamline 4-ID-C of the Advanced Photon Source in a liquid helium cryostat with powder samples mounted on carbon tape onto a cold finger in a 7 T (maximum) field magnet. Spectra were collected in total electron yield mode and XMCD was obtained by reversing the photon helicity at each energy interval. Spectra were measured at 10 K and 75 K in an applied field of 1 T. XMCD spectra presented are an average of the positive and negative field polarity measurements. All XMCD spectra were normalized to the maximum XAS intensity. Experimental spectra were modeled using ligand field multiplet calculations based on the method of van der Laan and Thole^37^ using the CTM4XAS program^38^.

### Magnetometry

Magnetization curves were measured for dilute (~0.1 vol. %) dispersions of core/shell in eicosane (melting point 42 °C), using a vibrating sample magnetometer (VSM). The magnetometry system used in this work is a from Quantum Design, Inc., physical property measurement system (PPMS) with vibrating sample magnetometry (VSM) option (Any mention of commercial products is for information only; it does not imply recommendation or endorsement by NIST). 9 T field-cooled hysteresis loops were recorded at 10 K to reveal evidence of exchange bias. Magnetization loops were measured at 5, 60, 200, and 300 K, at fields up to 9 T. The zero field-cooled magnetization was measured as a function of increasing temperature at 100 Oe. Dense assemblies were also measured, and were prepared by rapidly precipitating nanoparticles in gel capsules with the addition of 50% (by volume) ethanol. Ethanol and toluene were evaporated overnight leaving behind a dense assembly of particles. Due to rapid precipitation, these did now have the same long range stacking order (between particles) described below.

### Polarization-Analyzed Small-Angle Neutron Scattering (PASANS)

Polarization-Analyzed Small-Angle Neutron Scattering (PASANS) measurements were performed at the NIST Center for Neutron Research on the NG7 beamline, using 5.5 Å wavelength neutrons with a spread of 12%. For these measurements, the nanoparticles were first assembled into 3D crystals through slow precipitation as described previously^50,51^; the supracrystals were then dried to a powder, placed in aluminum foil and sealed in aluminum sample holders. The neutrons were polarized in a double-V super mirror cavity and flipped when desired using a radio-frequency flipper. The dense assembly of nanoparticles was cooled in a closed-cycle helium cryostat with an electromagnet used to apply magnetic fields up to 1.5 T. After scattering off the nanoparticles, the neutrons were spin-analyzed using a polarized ^3^He filter. The time-dependent polarization and spin leakage associated with the ^3^He filter were accounted for in the corrected data using a process described previously^41,42^; special care was taken to match the degree of polarization correction in the vicinity of the Bragg reflection to that observed in lower Q scattering regions. The corrected data for all four cross-sections were then analyzed using methods described previously^7,41,42^ and outlined in more detail in Data Note S5.

### Simulations

The nanoparticles were modelled as an idealised Fe_3_O_4_ core - MnFe_2_O_4_ shell structure approximated as a single crystal. The magnetic properties were modelled at the atomistic level using the VAMPIRE software package^52^ with Heisenberg exchange between atomic sites. The exchange parameters for the core and shell were taken the same as bulk Fe_3_O_4_ and bulk MnFe_2_O_4_ respectively, while the Dzyaloshinskii-Moriya interactions were calculated taking into account the local Oxygen symmetry about each ion based on the phenomenological model of Moriya^46^. The atomistic spin dynamics of the particles are simulated using numerical integration of the stochastic Landau-Lifshitz-Gilbert equation applied at the atomic level with critical Gilbert damping *α* = 1 to ensure a rapid convergence to the equilibrium spin state. Further details are provided in the Supplemental Information in Data Note S6.

### Data and materials availability

All data needed to evaluate the conclusions in the paper are present in the paper and/or the Supplementary Materials. Additional data related to this paper may be requested from the authors.

## Electronic supplementary material

Supplementary Information
