# Supplementary material for: Spin canting across core/shell Fe3O4/MnxFe3−xO4 nanoparticles
Source: Sci Rep. 2018 Feb 21;8:3425. doi: 10.1038/s41598-018-21626-0 (PMC5821856; doi:10.1038/s41598-018-21626-0)
Supplement: Supplementary file 1 — Supplementary Information [file 41598_2018_21626_MOESM1_ESM.pdf]

Supplemental information: Spin canting across core/shell $Fe_3O_4/Mn_xFe_{3-x}O_4$ nanoparticles

*Samuel D. Oberdick[1,2], Ahmed Abdelgawad[1,3], Carlos Moya[1], Samaneh Mesbahi-Vasey[4], Demie Kepaptsoglou[5], Vlado K. Lazarov[6], Richard F. L. Evans[6], Daniel Meilak[6], Elizabeth Skoropata[7], Johan van Lierop[7], Ian Hunt-Isaak[8], Hillary Pan[8], Yumi Ijiri[8], Kathryn L. Krycka[9], Julie A. Borchers[9], Sara A. Majetich[1]**

[1] Physics Department, Carnegie Mellon University, Pittsburgh, PA 15213, USA

[2] Applied Physics Division, Physical Measurement Laboratory, NIST, Boulder, CO 80305, USA

[3] Materials Science and Engineering Department, Carnegie Mellon University, Pittsburgh, PA 15213, USA

[4] Chemistry Department, Carnegie Mellon University, Pittsburgh, PA 15213, USA

[5] SuperSTEM, Sci-Tech Daresbury Campus, Daresbury, WA4 4AD, UK

[6] Department of Physics, University of York, Heslington, York, YO10 5DD, UK

[7] Physics and Astronomy Department, University of Manitoba, Winnipeg, MB R3T 2N2 Canada

[8] Physics and Astronomy Department, Oberlin College, Oberlin, OH 44074, USA

[9] NIST Center for Neutron Research, NIST, Gaithersburg, Maryland 20899, USA

*E-mail: sara@cmu.edu

Figure S1. Gallery of HAADF-STEM images. (a) Arrows point towards particles aligned along the [100] zone axis. Crystallographic surface disorder is also clearly observable. (b) Nanoparticle aligned along the [112] zone axis.

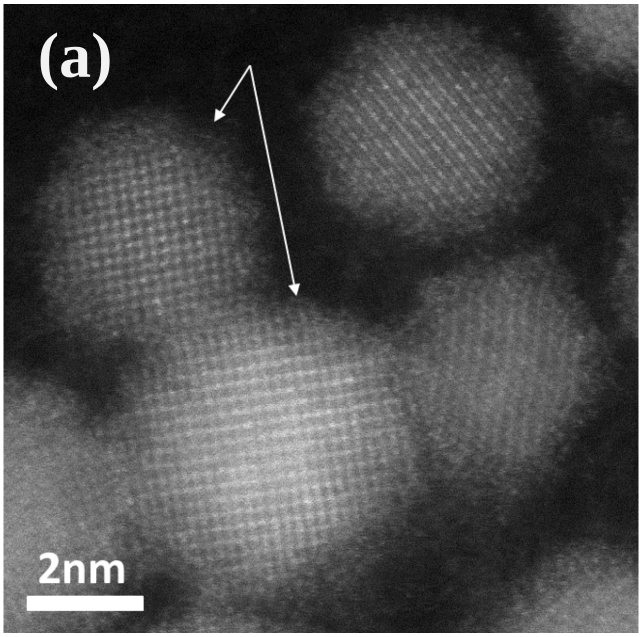

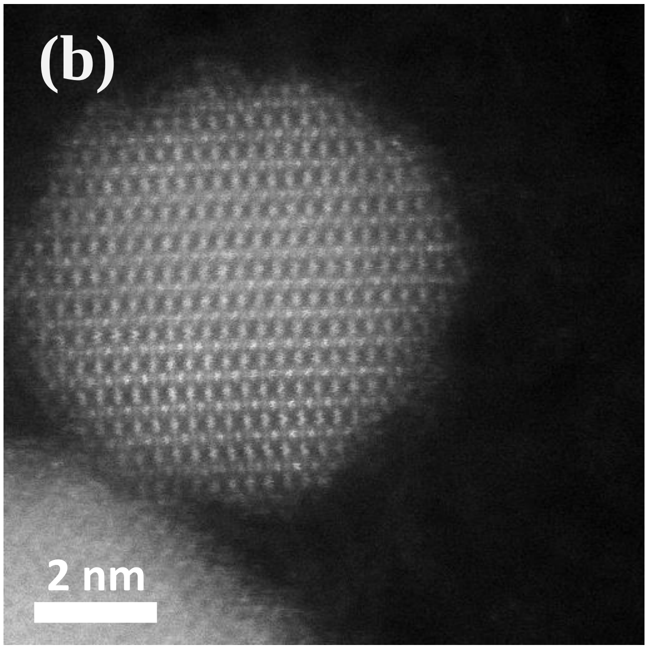

Table S2. Hyperfine parameters for the components of the Mössbauer spectrum measured at 10 K, where δ is the isomer shift, B_{hf} is the hyperfine field, Δ is the quadrupole splitting, and Γ is the line width.

Site	δ (mm/s)	B_{hf} (T)	Δ (mm/s)	Γ (mm/s)	Area (%)
I - Fe^{3+} A	0.549(5)	53.15(6)	0.026(9)	0.19(1)	26(7)
II – Fe^{3+} B	0.492(5)	51.78(7)	0.020(9)	0.22(2)	43(13)
III – Fe^{2+} B	0.520(8)	50.2(2)	-0.08(2)	0.26(2)	26(9)
IV – Fe^{2+} B	0.89(2)	44.6(4)	0	0.33(8)	5(3)

Figure S3. (a) Comparison of magnetization curves for a dilute dispersion of the NPs at 10, 200, and 300 K. (b) Magnified region around zero applied field.

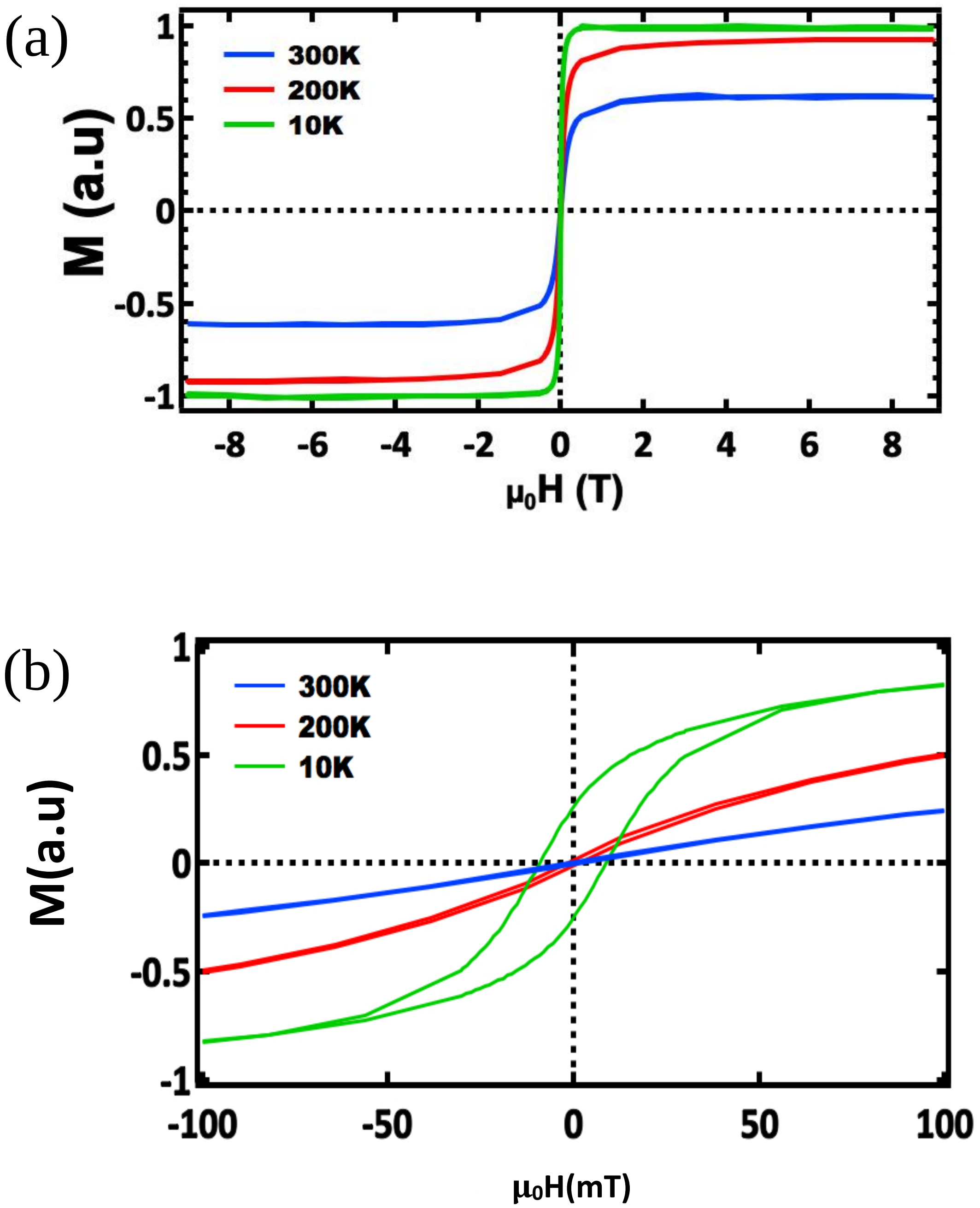

Figure S4. Field cooled (FC) and zero field cooled (ZFC) magnetization for (a) a dilute sample of the core/shell NPs. (b) Overlay of ZFC from both dilute and dense samples of nanoparticles. The blocking temperature of the particles in the dilute particles is lower than the dense samples, indicating that interparticle interactions have been reduced. The peak associated with blocking temperature is also much more distinct for dilute particles (fall off with increasing temperature is more abrupt) indicating that the dilute sample has reduced interparticle interactions. Here the applied field was 100 Oe.

(a)

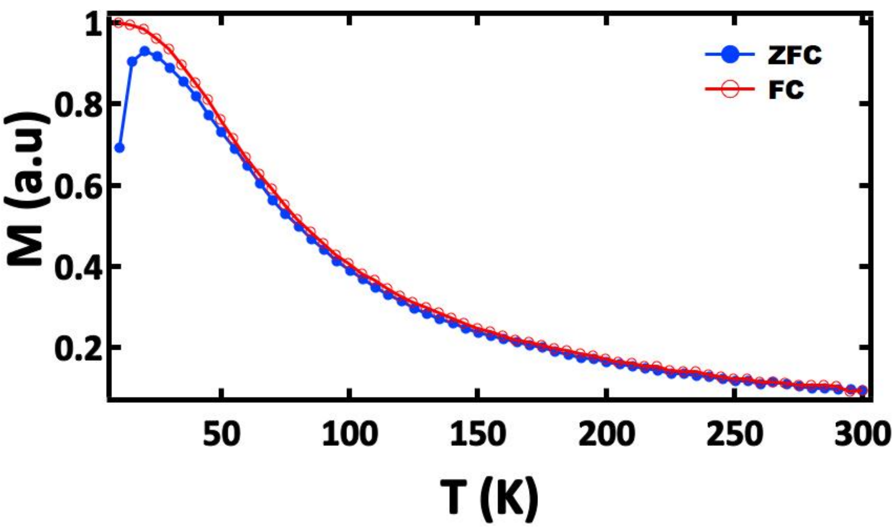

(b)

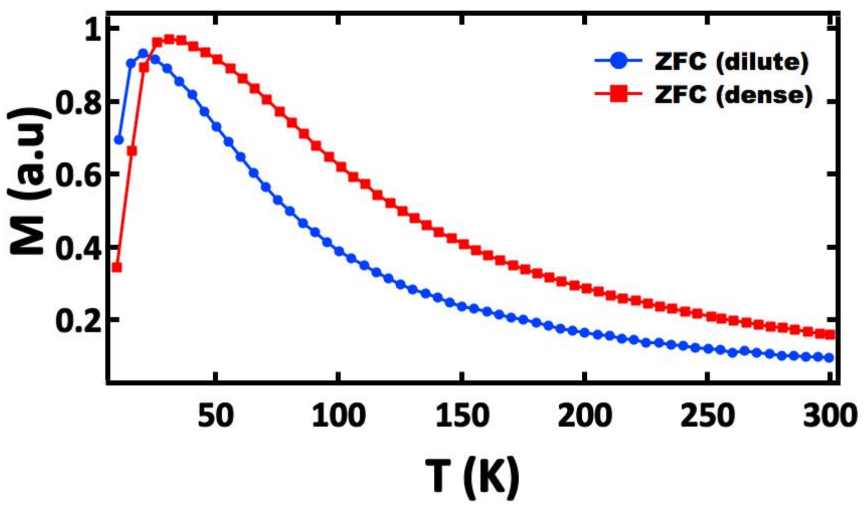

Data Note S5. Using the PASANS method, we obtained data for all four possible scattering cross-sections (↑↑, ↑↓, ↓↑, or ↓↓) corresponding to incident neutrons either spin up (↑) or spin down (↓) with the post-sample scattered neutrons, again either spin up (↑) or spin down (↓). For example, ↑↓, indicates the scattering from neutrons initially polarized spin up which then after scattering off the sample were found to polarized spin down; the scattering of this cross-section (↑↓) and the related one (↓↑) thus involve "spin-flipping" of the incident neutron relative to the scattered one.

These scattering cross-sections are proportional to the squared sum of the spatial nuclear N and magnetic M Fourier transforms[1,2]:

$$N, M_J(\boldsymbol{Q}) = \sum_K \rho_{N,M_J}(K)\exp(i\boldsymbol{Q}\cdot\boldsymbol{R}_K)$$

where J is any Cartesian coordinate (X, Y, or Z), **Q** is the scattering vector, $\mathbf{R}_K$ is the relative position of the Kth scatterer and $\rho_{N,M}$ is the nuclear or magnetic scattering length density respectively. Note that the nuclear scattering is assumed isotropic in many cases, although in some systems, nuclear spins can be aligned. In contrast, the magnetic Fourier transform has directional components with selection rules governing the observed scattering; only the component of magnetic scattering perpendicular to **Q** can contribute.

While in general the complete angle-dependent polarization rules lead to complex expressions for the scattering cross-sections, these simplify in certain geometries and key angles. In the present case, we then extract the quantities N^2, M^2_{PAR} and M^2_{PERP} in **Fig. 6b** from the underlying four cross-sections in the following way, taking sector averages of the data for the specified values of $\theta=0^o$or 90°, with sector widths of $\pm 10^o$ to obtain appropriate statistics:

$$N^2 \equiv |N(\boldsymbol{Q})|^2 = \uparrow\uparrow(|\boldsymbol{Q}|,0°)+\downarrow\downarrow(|\boldsymbol{Q}|,0°)$$

$$M^2_{PERP} \equiv |M_Z(\boldsymbol{Q})|^2 = \uparrow\downarrow(|\boldsymbol{Q}|,90°)+\downarrow\uparrow(|\boldsymbol{Q}|,90°)$$

$$M^2_{PAR} \equiv |M_X(\boldsymbol{Q})|^2 = \frac{[\downarrow\downarrow(|\boldsymbol{Q}|,90°)-\uparrow\uparrow(|\boldsymbol{Q}|,90°)]^2}{4[\uparrow\uparrow(|\boldsymbol{Q}|,0°)+\downarrow\downarrow(|\boldsymbol{Q}|,0°)]}$$

In the expression for N^2, we note that the sample is isotropic in our case, given that the polycrystals of ordered nanoparticle assemblies do not have particular preferred directions. The expression for M^2_{PERP} is explicitly for a particular portion of the spin-flip data, while M^2_{PAR} equation is for the fraction that is coherent with the structural order.

Data Note S6. Details of atomistic simulations. The structure of the measured nanoparticles is complex and so we have adopted a simplified model based on a single crystal particle to capture the essential properties of the particles. We construct a single crystal of Magnetite with an inverse Spinel structure and lattice parameter 8.3941 Å, explicitly including the Oxygen sites due to their contribution to the DMI on octahedral Mn sites. A spherical particle is then cut from the crystal and different magnetic parameters are assigned based on the distance from the centre, defining a core region 5.6 nm in diameter and a shell of 0.7 nm thick. The magnetic properties of the system are described with a Heisenberg spin Hamiltonian of the form

$$\mathcal{H} = -\sum_{i<j} \boldsymbol{S}_i \boldsymbol{J}_{ij}^T \boldsymbol{S}_j - \sum_i \mu_i \boldsymbol{H}_{\text{app}}$$

where $\boldsymbol{S}_i$ and $\boldsymbol{S}_j$ are unit vectors describing the directions of spins on sites i and j, $\boldsymbol{J}_{ij}^T$ is the exchange tensor between sites i and j, μ_i is the spin moment at site i and $\boldsymbol{H}_{\text{app}}$ is a vector describing the direction of the externally applied field. The exchange tensor describes isotropic, anisotropic and anti-symmetric (DMI) exchange interactions between two spin sites i and j and is given by

$$\boldsymbol{J}_{ij}^T = \begin{bmatrix} J_{xx} & D_z & -D_y \\ -D_z & J_{yy} & D_x \\ D_y & -D_x & J_{zz} \end{bmatrix}$$

where $J_{xx} = J_{yy} = J_{zz} = J_{ij}$ is the usual isotropic exchange interactions, and D_x, D_y and D_z are the directional components of the DMI given by[3]

$$\boldsymbol{D}_{ij} = D\, \boldsymbol{r}_{ik} \times \boldsymbol{r}_{jk}$$

where atom k is a mediating Oxygen atom between magnetic sites i and j. Since the DMI strength is proportional to the Mn-O-Mn bond angle the strongest interactions are naturally between

octahedral B-sites due to the 78° bond angle. The magnetic moments are assigned as $Mn_{3+} = 5\ \mu_B$, $Fe^{T}_{3+} = 5\ \mu_B$ and $Fe^{O}_{2.5+} = 4.5\ \mu_B$ reflecting the mixed valence and half metallicity of the octahedral Fe B sites.

The atomistic spin dynamics are computed numerically by solving the stochastic Landau-Lifshitz-Gilbert equation with Langevin dynamics[4] applied at the atomic level using the VAMPIRE software package [5]. The simulations were performed with critical Gilbert damping $\alpha = 1$ to ensure a rapid convergence to an equilibrium spin state.

Figure S7. Visualization of the simulated spin configuration of a decoupled superparamagnetic $MnFe_2O_4$ shell taking a slice through the *y*-*z* plane. The coloring of the atoms indicates the direction of magnetization on each site, with Oxygen sites shown as small dark spheres. The simulation temperature is set at 300K in a 1 T externally applied field along the [001] crystal direction. The shaded core region shows a nearly single domain state, while the shell shows much more disorder due to finite size effects. At high fields, the superparamagnetic shell is well aligned with the field direction leading to a Langevin-type saturation of the total magnetization M_s for the nanoparticle.

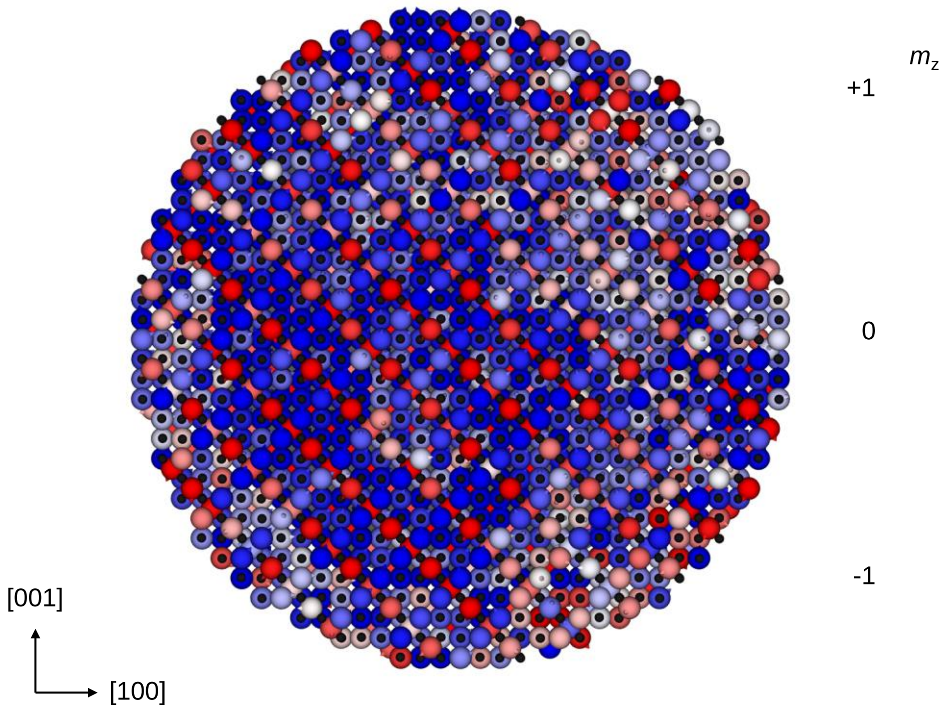

Figure S8. *M(H)* loop at 300 K for a dense assembly. The loop does not saturate entirely up to 9 T. The sample still has susceptibility at high fields.

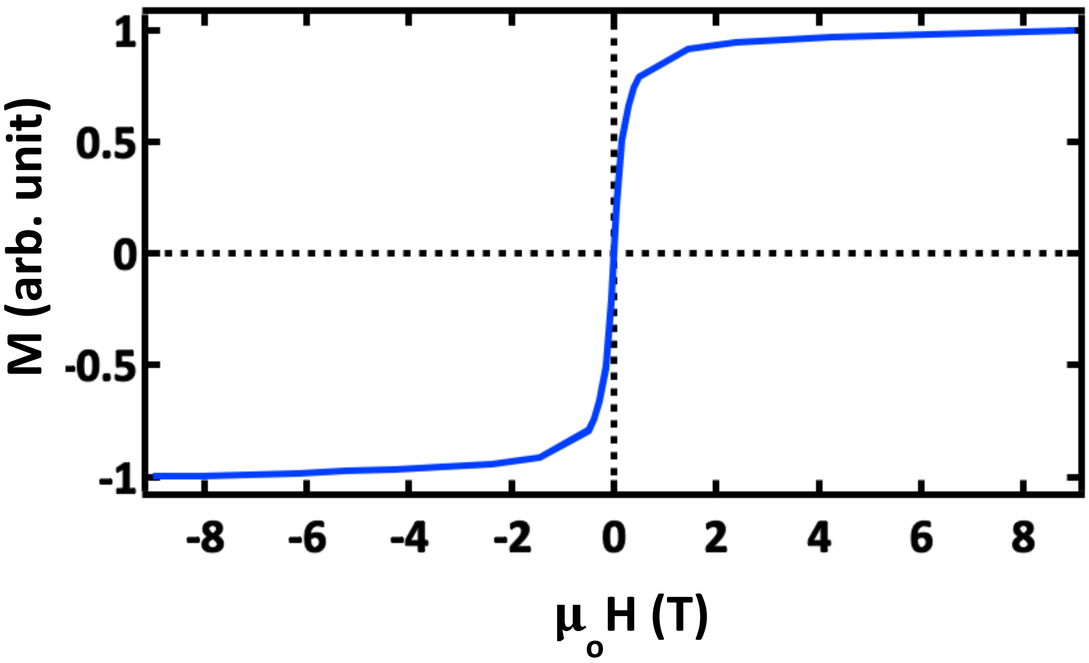

Figure S9. Background subtracted EELS core loss spectra from the core and shell regions of the $Fe_3O_4/(Mn,Fe)_3O_4$ cores/shell NPs, showing the Mn and Fe $L_{2,3}$ ionization.

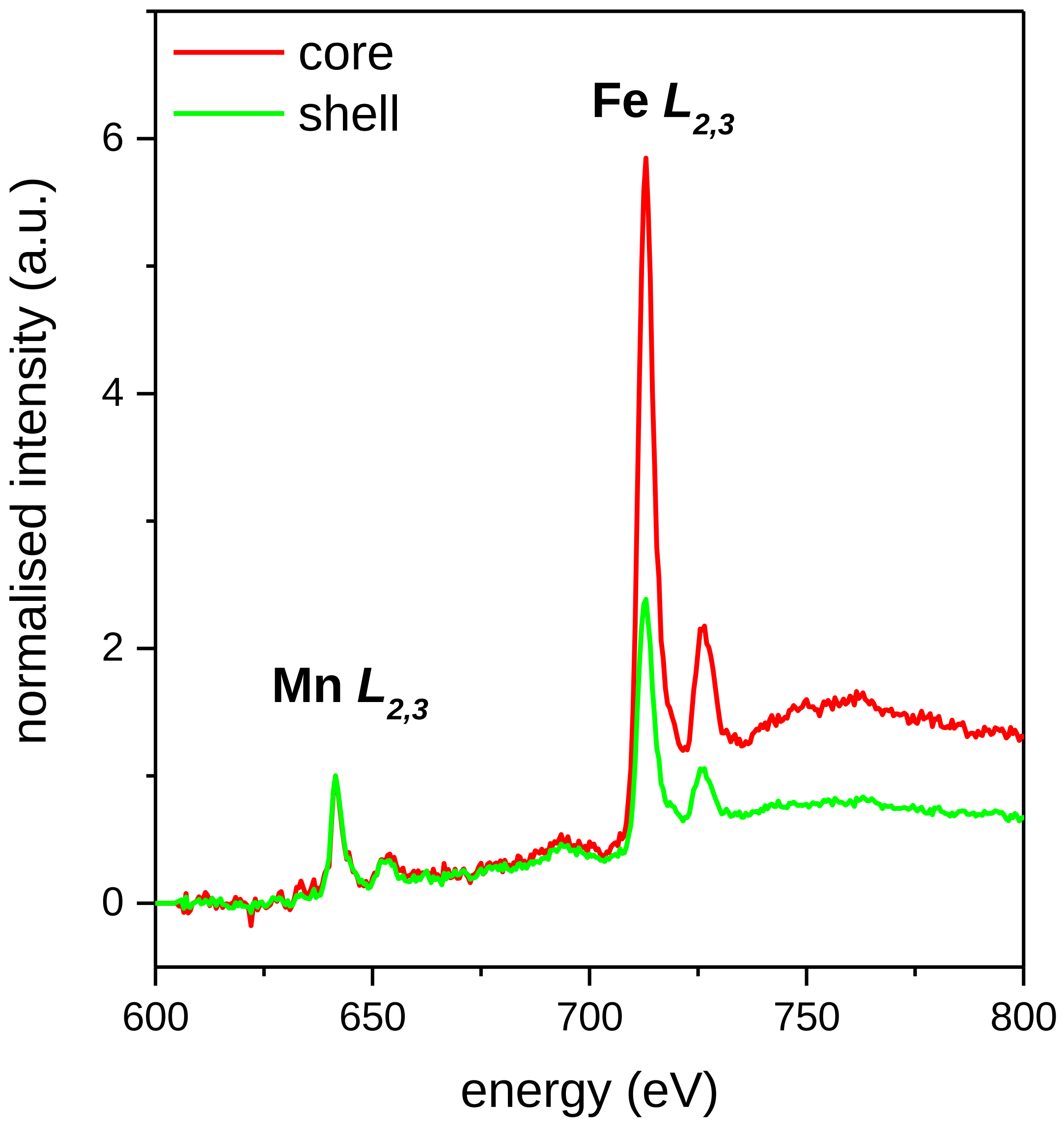

References

1. Krycka, K., Chen, W., Borchers, J., Maranville, B. & Watson, S. Polarization-analyzed small-angle neutron scattering. I. Polarized data reduction using Pol-Corr. *J. Appl. Crystallogr.* **45,** 546–553 (2012).
2. Krycka, K., Borchers, J., Ijiri, Y., Booth, R. & Majetich, S. Polarization-analyzed small-angle neutron scattering. II. Mathematical angular analysis. *J. Appl. Crystallogr.* **45,** 554–565 (2012).
3. Moriya, T. Anisotropic Superexchange Interaction and Weak Ferromagnetism. *Phys. Rev.* **120,** 91–98 (1960).
4. Evans, R. F. L. *et al.* Atomistic spin model simulations of magnetic nanomaterials. *J. Phys. Condens. Matter* **26,** 103202 (2014).
5. *VAMPIRE software package 5.0 available from http://vampire.york.ac.uk. R F L Evans et al.*
